# BetaCavityWeb: a webserver for molecular voids and channels

**DOI:** 10.1093/nar/gkv360

**Published:** 2015-04-22

**Authors:** Jae-Kwan Kim, Youngsong Cho, Mokwon Lee, Roman A. Laskowski, Seong Eon Ryu, Kokichi Sugihara, Deok-Soo Kim

**Affiliations:** 1 Vorononi Diagram Research Center, Hanyang University, Korea; 2 School of Mechanical Engineering, Hanyang University, Korea; 3 European Bioinformatics Institute, Wellcome Trust Genome Campus, UK; 4 Department of Bioengineering, Hanyang University, Korea; 5 Meiji Institute for Advanced Study of Mathematical Sciences, Tokyo, Japan

## Abstract

Molecular cavities, which include voids and channels, are critical for molecular function. We present a webserver, *BetaCavityWeb*, which computes these cavities for a given molecular structure and a given spherical probe, and reports their geometrical properties: volume, boundary area, buried area, etc. The server's algorithms are based on the Voronoi diagram of atoms and its derivative construct: the beta-complex. The correctness of the computed result and computational efficiency are both mathematically guaranteed. BetaCavityWeb is freely accessible at the Voronoi Diagram Research Center (VDRC) (http://voronoi.hanyang.ac.kr/betacavityweb).

## INTRODUCTION

Molecular structure determines molecular function. Of particular importance are the ‘gaps’ within a structure—internal voids, channels running through it, pockets in the surface—because biological molecules perform their tasks via interactions with other molecules in their environment and these interactions often take place in these spaces. This paper reports a webserver, BetaCavityWeb, which recognizes voids and channels in molecular structures and measures their geometrical properties such as volume, area of cavity boundary and atoms contributing to geometric features.

Here we define a void as a cavity in a molecular interior that is not accessible to bulk solvent around the molecule and can be either hydrated or free from any solvent molecule. A channel is defined as a hole penetrating a molecular structure with two or more openings toward the exterior space through which a solvent or ligand molecule can freely pass (1,2).

The first work on the properties of voids in molecular structures was Connolly's MS program in the early 80s (3,4). MS used a rolling ball on the surface of a molecule to define a shell corresponding to the external molecular boundary and an internal void boundary. Many methods have since been developed for identifying the empty regions, specifically for locating potential binding sites. In 1994, Kleywegt and Jones developed VOIDOO which recognized voids by embedding the molecule in a grid and checking which grid points were not within any of the atoms (5). Another method using a grid was AVP (6). The SURFNET program fitted spheres into the spaces between pairs of atoms and then identified the voids as clusters of these spheres (7). Sheffler and Baker developed RosettaHoles to recognize voids by first generating a set of void-filling balls that cover the interstitial space in a molecule and then applying the statistical learning technique of support vector machines (8). Liang *et al*. developed the VOLBL program using the alpha-shape which was based on the power diagram (9) and further improved into CASTp (10). Most recently the BetaVoid program was developed by Kim *et al*. and used the beta-complex derived from the Voronoi diagram of spherical atoms and its derivative constructs. They showed that it outperforms existing methods (11) including CASTp (10).

The earliest efforts on the recognition of molecular channels came during the mid 90s for computing the pore dimensions of ion channels. The HOLE program (12) required the user to specify an initial location within a channel and its direction. It then used Monte Carlo simulation to recognize the channel. Subsequent studies on channel recognition have been reported relatively recently. In 2006, Voss *et al*. showed that channel geometry is important for ribosomal polypeptides in determining biomolecular function (13). Channel geometry is also important for transmembrane proteins in selecting transport of ions, small molecules and even large molecules (14–18). In 2006, Damborský and colleagues reported a grid-based method for extracting cavities by tracing routes from buried active sites to the external solvent and developed CAVER (19) as a plug-in to PyMOL (20). Otyepka and colleagues developed MOLE using the ordinary Voronoi diagram of atom center points (21). The MolAxis program used the alpha-shape of the molecule, where atoms were approximated by a number of identically sized balls (22). In 2009, Pellegrini-Calace *et al*. reported the PoreWalker server which computed channels in transmembrane proteins. The method took advantage of the knowledge that secondary structures are usually aligned with the medial axis of the channel, and additionally used to geometric information (23). An important step forward was made by Hege and colleagues in 2011 (24) who adapted an algorithm which traced the edges in the Voronoi diagram of atoms (25). In 2012, Otyepka's group reported the MOLE*online* 2.0 server, which was an improved version of the original MOLE program mentioned above (26), and Damborský's group produced a new version of CAVER, as CAVER 3.0, which could analyze both static structures and molecular dynamic trajectories, now using the ordinary Voronoi diagram of points by approximating atoms with several spherical balls with identical radii (27).

There are two critical issues in cavity recognition. First, an accurate, efficient and convenient approach is necessary for recognizing and measuring cavities. Vlassi *et al*. have pointed out the difficulty of comparing void sizes produced from different programs due to the different computational methods and parameters used (28). They concluded that cavity volume was a poor parameter for analyzing structural responses to mutation because of the unreliable and somewhat contradictory computational results. This suggests that a more accurate and reliable mathematical method and its implementation is necessary. Second, there are various types of information to be analyzed and reported regarding cavities. The volume and area are only two of these. For example, Hubbard *et al*. pointed out that there were numerous types of analysis possible regarding molecular voids (29): position, size and shape, number of voids, polarity, packing of solvent in cavities, distribution of cavity volumes for solvated and empty cavities, amino acid preferences for accessible and buried protein surfaces, hydrogen bonding of polar atoms and solvent within voids, mobility of cavity atoms, etc. For this purpose, it is necessary to have a formal representation of molecular structure. These two issues are the motivation for BetaCavityWeb. Voids and channels are indeed important quality measures of computational protein design (30). BetaCavityWeb is accurate, efficient and convenient and is freely available at the VDRC (http://voronoi.hanyang.ac.kr/betacavityweb).

## METHODS AND MATERIALS

### Voronoi diagrams, quasi-triangulations and beta-complexes

**Figure 1.**
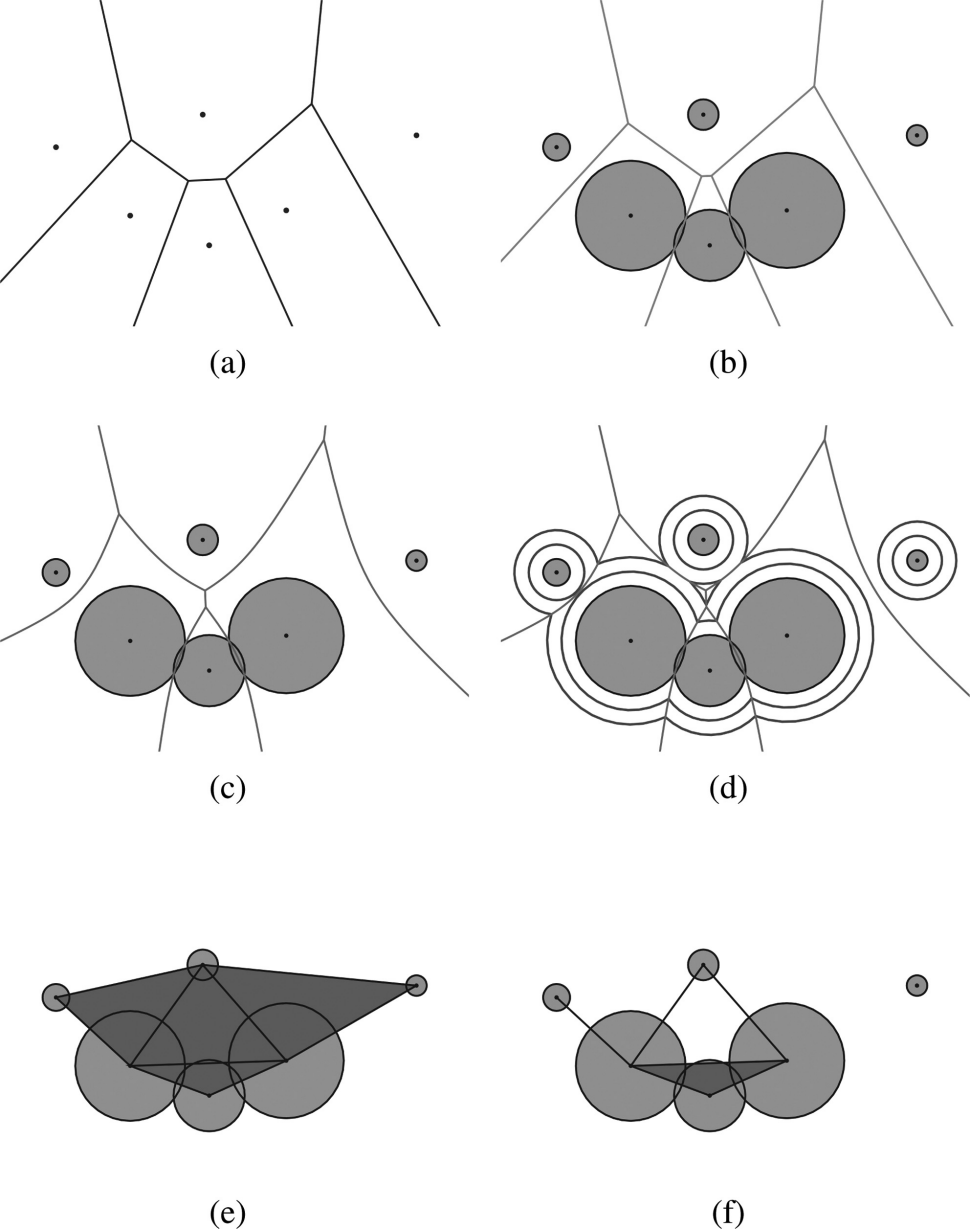
Voronoi diagrams and derivative constructs (figures drawn by the BetaConcept program). (**a**) Ordinary Voronoi diagram of center points of circle generators, (**b**) power diagram of circle generators, (**c**) Voronoi diagram of circle generators, (**d**) invariant Voronoi diagram of offset, (**e**) quasi-triangulation and (**f**) beta-complex.

Let *A* = {*a*_*1*_, *a*_*2*_, …, *a*_*n*_} be a set of three-dimensional spherical atoms where *a*_*i*_ = (*c*_*i*_, *r*_*i*_) is an atom with center *c*_*i*_ and vdW-radius *r*_*i*_. Consider *A* a molecule. Let $\mathcal {VC}(a_i)$ be the Voronoi cell for *a*_*i*_ defined as $\mathcal {VC}(a_i) = \lbrace x \in \mathbb {R}^3 | d(x, c_i)-r_i \le d(x, c_j) - r_j, i \ne j \rbrace$. Then, the Voronoi diagram $\mathcal {VD}$ for the atom set *A* is defined as $\mathcal {VD} = \lbrace \mathcal {VC}(a_1), \mathcal {VC}(a_2), \cdots , \mathcal {VC}(a_n) \rbrace$ where the connectivity among the topological entities are appropriately represented. In the three-dimensional space, $\mathcal {VD}$ can be represented as $\mathcal {VD}=(V^\mathcal {V},E^\mathcal {V},F^\mathcal {V},C^\mathcal {V})$ where $V^\mathcal {V}$ is the set of Voronoi vertices, $E^\mathcal {V}$ is the set of Voronoi edges, $F^\mathcal {V}$ is the set of Voronoi faces and $C^\mathcal {V}$ is the set of Voronoi cells. The topology among vertices, edges, faces and cells in $\mathcal {VD}$ are properly maintained in the radial-edge data structure (31). Suppose that *A*^*O*^ is an offset model where $a^O_i \in A^O$ is an offset atom where its radius is increased by a constant amount *δ* from the atom *a*_*i*_ ∈ *A*. Then, it is known that the topology of the Voronoi diagram $\mathcal {VD}^O$ for *A*^*O*^ is identical to that of $\mathcal {VD}$. Thus, we call the Voronoi diagram of atoms $\mathcal {VD}$ offset-invariant. Formally, in the computational geometry community, $\mathcal {VD}$ is called the additively weighted Voronoi diagram. Note that $\mathcal {VD}$ in Figure 1(c) is different from both the ordinary Voronoi diagram of points where the points correspond to atom centers in Figure 1(a) and the power diagram in Figure 1(b). For the details of $\mathcal {VD}$ and its algorithm, refer to (25,32) and for the Voronoi diagram in general, refer to (33). Figure 1(c) shows the $\mathcal {VD}$ of a two-dimensional atom set *A* which consists of six circular disks. Figure 1(d) shows the two offset curves for different offset amounts where the intersections between offset circles are always placed on the Voronoi edges.

The quasi-triangulation $\mathcal {QT}$ is the dual structure of $\mathcal {VD}$ and is represented as $\mathcal {QT}=(V^\mathcal {Q},E^\mathcal {Q},F^\mathcal {Q},C^\mathcal {Q})$ where $v^\mathcal {Q} \in V^\mathcal {Q}$, $e^\mathcal {Q} \in E^\mathcal {Q}$, $f^\mathcal {Q} \in F^\mathcal {Q}$ and $c^\mathcal {Q} \in C^\mathcal {Q}$ are one-to-one dual-mapped from $c^\mathcal {V} \in C^\mathcal {V}$, $f^\mathcal {V} \in F^\mathcal {V}$, $e^\mathcal {V} \in E^\mathcal {V}$ and $v^\mathcal {V} \in V^\mathcal {V}$, respectively. As $\mathcal {VD}$ is different from the ordinary Voronoi diagram of points, $\mathcal {QT}$ is different from the Delaunay triangulation which is the dual structure of the ordinary Voronoi diagram. $\mathcal {VD}$ is offset-invariant and so is $\mathcal {QT}$. For the details of $\mathcal {QT}$, see (34–36). Figure 1(e) shows $\mathcal {QT}$ of *A*.

The beta-complex $\mathcal {BC}$ is defined from $\mathcal {QT}$ when a spherical probe with radius β is given. Consider an edge *e* in $\mathcal {QT}$ and the two atoms corresponding to the vertices of *e*. If the probe can pass between the two atoms without any intersection, we remove *e* from $\mathcal {QT}$. Similarly, if the probe can pass among the three atoms corresponding to the vertices of a triangular face *f* in $\mathcal {QT}$, we remove *f* from $\mathcal {QT}$. If we apply this operation for all the simplexes in $\mathcal {QT}$, we eventually have the beta-complex $\mathcal {BC}$ which is therefore a subset of $\mathcal {QT}$. The region of Euclidean space bounded by $\mathcal {BC}$ is called the beta-shape $\mathcal {BS}$. Hence, each simplex on the boundary of $\mathcal {BS}$ determines the proximity among the atoms on the boundary of the molecule where the boundary is defined by the probe. Each simplex in $\mathcal {BC}$ determines the proximity among all atoms on and within the boundary of the molecular structure. We emphasize here that the beta-complex can be computed very efficiently from the quasi-triangulation. For details, see (37). Figure 1(f) shows an example of $\mathcal {BC}$ for *β*-value. Note that Figure 1 was drawn by BetaConcept (38).

### The BetaCavity algorithm

Consider a vdW-molecule *A* and its offset model *A*^*O*^, offset by an amount *δ* ≥ 0. The boundary ∂*A*^*O*^ of the offset model is equivalent to the Lee–Richards (solvent accessible) surface for a solvent molecule probe with the radius *δ*.

Suppose that $\mathcal {VD}=(V^\mathcal {V},E^\mathcal {V},F^\mathcal {V},C^\mathcal {V})$ of *A* is computed. Due to the offset invariant property, $\mathcal {VD}$ is also the Voronoi diagram of *A*^*O*^. Let $Vor=(V^\mathcal {V},E^\mathcal {V},F^\mathcal {V})$ be an abstraction of $\mathcal {VD}$ without V-cells. We compute the Voronoi complement *Vor*^*C*^ = (*V*^*C*^, *E*^*C*^, *F*^*C*^) by trimming the structure of *Vor* with each offset atom in the offset model *A*^*O*^. *Vor*^*C*^ can be computed in *O*(*m*) time in the worst case, where *m* represents the number of entities in the Voronoi diagram. The BetaCavity algorithm is detailed in (39) from which we quote the following theorem fundamental for the BetaCavityWeb server. Note that the BetaVoid program is the implementation for voids (11).

Theorem 1. *The Voronoi complement Vor*^*C*^ *and the space external to an offset model are homotopy equivalent*.

Therefore, a Voronoi complement and the exterior of an offset model have an identical topological property and its structure can be used to correctly recognize the voids and channels of the offset model. A Voronoi complement consists of one or more components. If there is only one component, it corresponds to the external space possibly with channels without any void. If there are two or more components, the one connected to infinity corresponds to the external space and each of the others corresponds to a void. Given *Vor*^*C*^ = {*ξ_1_, ξ_2_*, …} where *ξ_i_* is a component, we classify its components corresponding to the unbounded external region and voids.

There are two types of Voronoi edges in the component *ξ* of *Vor*^*C*^ for the unbounded region: those intersecting the boundary of an offset model and those non-intersecting. Similarly, there are two types of Voronoi faces: those intersecting and those non-intersecting Voronoi faces. Given *ξ*, we remove the intersecting edges and faces. Then, the contraction of each Voronoi face to one of its bounding Vornoi edges reduces *ξ* to a graph called the Voronoi graph which consists of vertices and edges. Then, the Euler-Poincaré formula of the Voronoi graph of *ξ* gives the information about the topology of the channel (i.e. the existence of handles). In the case of channels, the Voronoi graph is called its spine and reveals the topology of the channel. For details about the Euler-Poincaré formula, see (31). The volume and area of each cavity can be correctly and efficiently computed by applying the beta-decomposition algorithm (40). The Supplementary material illustrates the recognition of channels and voids using two example molecules.

## RESULTS AND DISCUSSION

### The functions of the BetaCavityWeb server

BetaCavityWeb has two ways of taking an input molecular structure. As the server mirrors the molecular structures in the PDB (41), users can simply enter a PDB accession code. Alternatively, users may upload their own molecular structures stored in PDB format. Users can indicate whether they wish to compute voids and/or channels with respect to a solvent probe with a specified radius, β.

**Figure 2.**
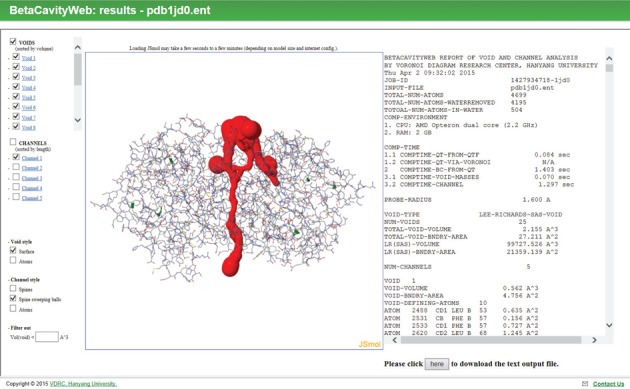
An output page for human carbonic anhydrase XII (PDB id: 1jd0).

BetaCavityWeb produces both text and graphics output as shown in Figure 2 (For 1jd0, see (42); For the role of the channel in carbonic anhydrase, see (43)). The textual output consists of three sections: the header, containing overall information about the session, and two other sections containing information on the voids and channels in the structure. Statistics such as the van der Waals volume and area and the computation time are also reported. Each of the void and channel sections contain information such as the number of cavities, their geometrical properties, a list of atoms defining their boundary, etc.

A probe radius of zero corresponds to the van der Waals surface and thus the recognized cavities are the van der Waals cavities. If the probe radius is nonzero, the recognized cavities are the Lee–Richards (solvent accessible) cavities. In the case of channels, BetaCavityWeb further reports the topological properties of each one: the number of openings (i.e. entrances and exits), the number of topological handles within the channel, the number of atoms contributing to the channel boundary, the atom triplets defining an area on the channel boundary, etc. BetaCavityWeb also reports channel spines and the bottleneck of each channel (i.e. its narrowest point). We emphasize that solution correctness and computational efficiency are mathematically guaranteed. BetaCavityWeb allows a user to choose individual cavities for further analysis. The textual output can be customized by the user before downloading.

**Figure 3.**
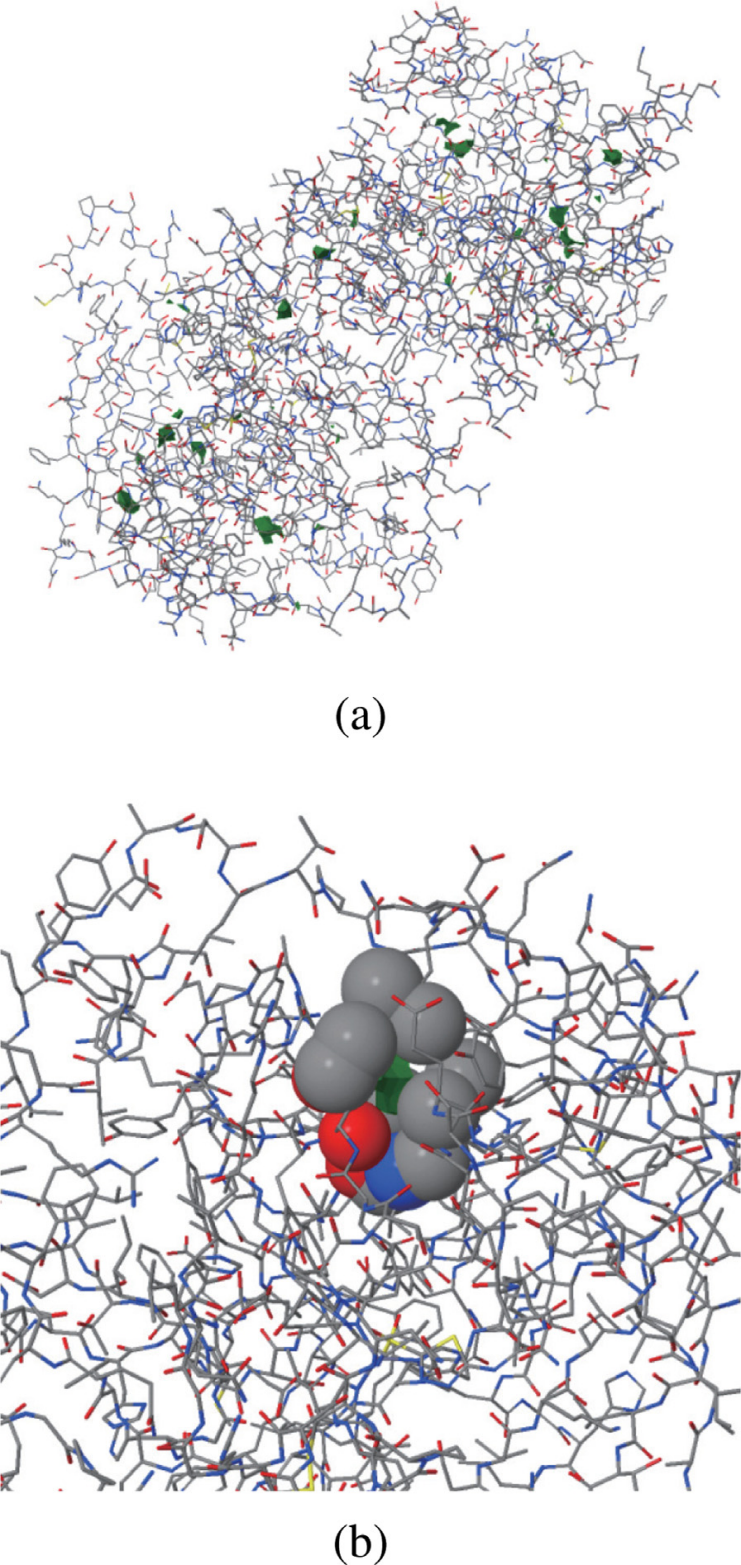
Voids (1jd0, probe radius: 1.4Å) computed by and visualized in BetaCavityWeb. (**a**) Lee–Richards (accessible) surface representation, and (**b**) contributing atoms of the biggest void.

**Figure 4.**
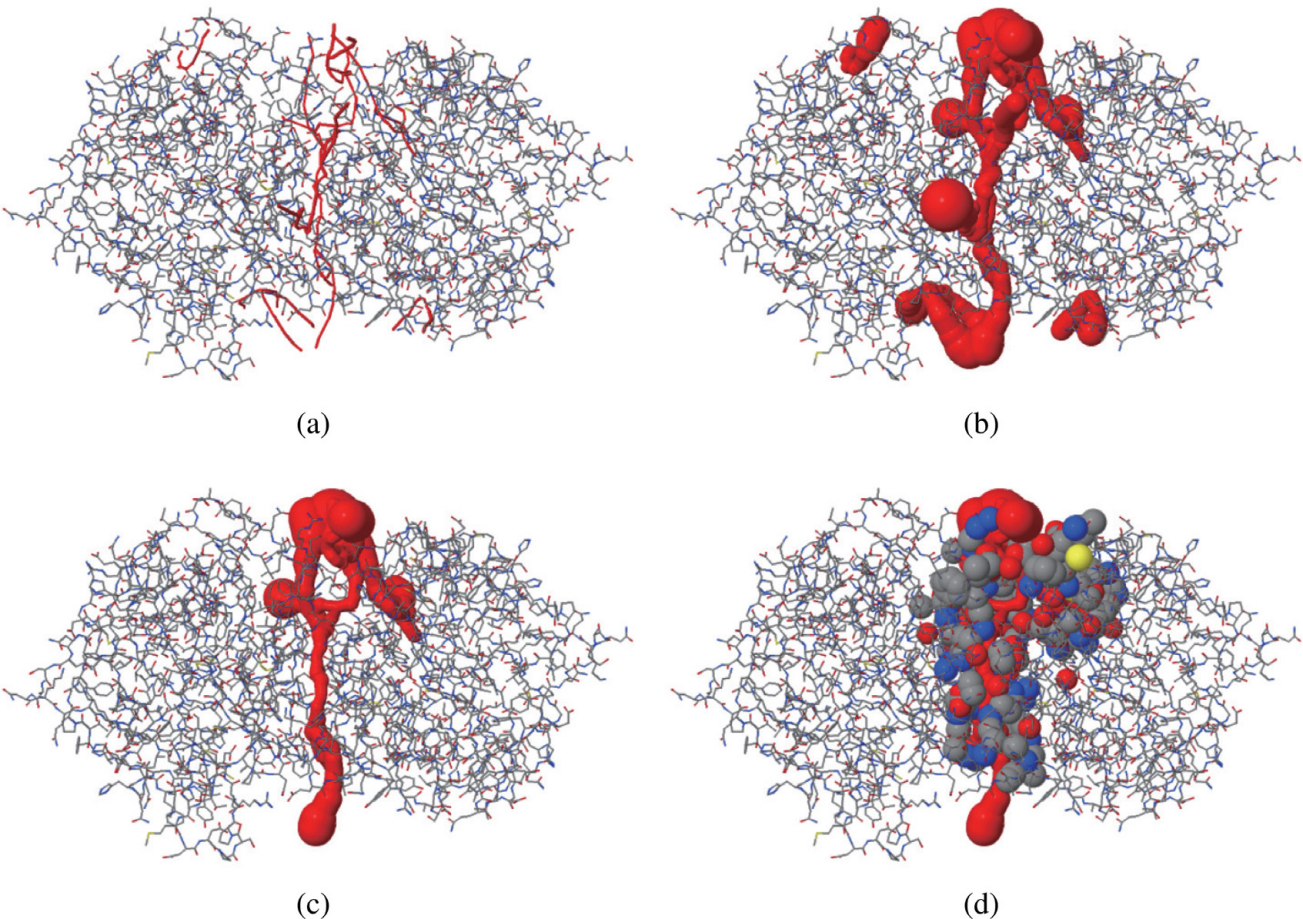
The channels for 1jd0 (probe radius: 1.4Å) computed by and visualized in BetaCavityWeb. (**a**) the spines of all recognized channels, (**b**) the spine-sweeping ball of all channels (Tiny ones are meaningless), (**c**) the spine-sweeping ball of the biggest channel and (**d**) the atoms contributing to the boundary of the biggest channel.

The graphical output, which employs JSmol (44), is shown in Figure 2. The computed voids and channels can be displayed together with a molecular structure represented by a space-filling, ball-and-stick, stick, or line model. Figure 3(a) and (b) show the computed voids in the Lee–Richards solvent accessible surface and the atoms contributing to the boundary of the largest void, respectively. Channels can be visualized in three different ways: (i) a spine, (ii) a radius-varying ball sweeping through a spine where the radius is determined by the perpendicular distance from its center to the boundary of nearby atoms and (iii) the atoms contributing to channel boundary. Figure 4 shows channels: (a) the spines of all recognized channels, (b) the spine-sweeping ball of all channels, (c) the spine-sweeping ball of the largest channel and (d) the atoms contributing to the boundary of the largest channel. The largest channel is usually meaningful from a biological point of view while the tiny ones, such as those in Figure 4(b), are meaningless as they are defined only from a geometric point of view.

### The components of BetaCavityWeb

**Figure 5.**
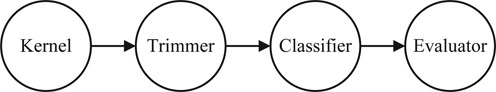
The BetaCavityWeb server's components and computational flow.

BetaCavityWeb is composed of four components: (i) a geometric kernel, (ii) a trimmer of the Voronoi diagram, (iii) a classifier of the Voronoi graph and (iv) an evaluator of geometric properties. The geometric kernel computes the Voronoi diagram, transforms to a quasi-triangulation and extracts the beta-complex. The trimmer computes the Voronoi complement by trimming the Voronoi structure intersecting a van der Waals molecule or a Lee–Richards solvent accessible surface model (i.e. the offset model) and converts the Voronoi complement into the Voronoi graph. The classifier parses the Voronoi graph to recognize voids and channels. The evaluator computes geometrical properties such as volume, boundary area, etc. of the recognized voids and channels. Figure 5 shows how these components are related: arrows denote the computational logic and data flow.

### QTDB: computational acceleration

**Figure 6.**
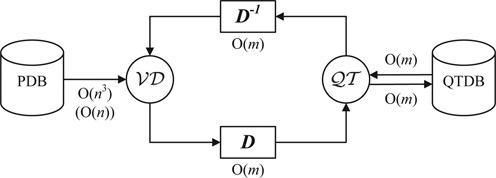
The computational acceleration with QTDB: *n*: # atoms; *m*: # simplexes; *m* = *O*(*n*^2^) for general spheres but *m* = *O*(*n*) for molecules.

The timing for the computation of the quasi-triangulation for molecular structures in the PDB is important. In most cases, a molecule of interest might have several analyses performed. In such cases the quasi-triangulation only needs to be computed once and can be reused for subsequent analyses. Hence, it is convenient to compute the quasi-triangulation in a preprocessing stage and store it in a database so it can be recalled when required. This approach is possible because the Voronoi diagram of atoms and the quasi-triangulation are offset invariant. For this purpose, we have defined a quasi-triangulation file format (QTF) (45) to store the data in a quasi-triangulation database (QTDB), available from the VDRC. Users can simply download the QTF file corresponding to the PDB file of interest and convert it to a Voronoi diagram. The QTF file takes *O*(*m*) memory for a quasi-triangulation with *m* simplexes and the conversion from a quasi-triangulation to the Voronoi diagram or vice versa takes *O*(*m*) time in the worst case. If it is necessary or desirable, users can build their own QTDB by running the QTFier program available from the VDRC. Figure 6 shows this approach.

## CONCLUSIONS

We report the BetaCavityWeb server which recognizes molecular voids and channels and computes their geometric properties. BetaCavityWeb is based on the Voronoi diagram of atoms, its quasi-triangulation and the beta-complex whose properties are all mathematically proven. The algorithms used in the computation are correct and efficient with mathematical guarantee. With the BetaCavityWeb server, researchers can easily and freely access the powerful capabilities of the Voronoi diagram of atoms to analyze molecular voids and channels.

## SUPPLEMENTARY DATA

Supplementary Data are available at NAR Online.

SUPPLEMENTARY DATA
