# Supplementary material for: BetaCavityWeb: a webserver for molecular voids and channels
Source: Nucleic Acids Res. 2015 Apr 22;43(Web Server issue):W413–8. doi: 10.1093/nar/gkv360 (PMC4489219; doi:10.1093/nar/gkv360)
Supplement: SUPPLEMENTARY DATA [file supp_43_W1_W413__index.html]

BetaCavityWeb: a webserver for molecular voids and channels — BetaCavityWeb: a webserver for molecular voids and channels — SUPPLEMENTARY DATA 

# BetaCavityWeb: a webserver for molecular voids and channels

## SUPPLEMENTARY DATA

**Files in this Data Supplement:**

- SUPPLEMENTARY DATA
