# Supplementary material for: BetaCavityWeb: a webserver for molecular voids and channels
Source: Nucleic Acids Res. 2015 Apr 22;43(Web Server issue):W413–8. doi: 10.1093/nar/gkv360 (PMC4489219; doi:10.1093/nar/gkv360)
Supplement: SUPPLEMENTARY DATA [file supp_gkv360_nar-00301-web-b-2015-File004.pdf]

Supplementary Material

Fig. S1(a) shows the Connolly surface of human carbonic anhydrase XII (PDB code: 1jd0) for a water molecule probe, usually represented by a sphere of radius 1.4Å. Note that there is a white spot in the middle implying the existence of a channel. Fig. S1(b) shows the recognized channels for water molecules where each color denotes a distinct channel. Fig. S1(c) shows both voids and channels. Fig. S1(d) shows the molecule from a different viewpoint with only the two most significant channels. Fig. S1(e) depicts the two channels with their spines, portraying the channel topology. Fig. S1(f) and S1(g) show the most significant channel and its spine; the channel bottleneck is depicted by the green ball centered at a point on one of its edges, respectively. The radius of the ball is the bottleneck radius of the spine. The holes with red boundaries on the channel surface in Fig. S1(e) and S1(f) denote the connection to neighbor channels where water molecules cannot pass through. Fig. S1(h) gives the Voronoi complement Vor^C from which the channel in Fig. S1(f) was computed.

Fig. S2(a) shows a space-filling representation of a proteasome (PDB code: 3shj, 50,877 atoms). The voids for a water probe are depicted in Fig. S2(b) and the channels for a probe radius of 2.3Å in Fig. S2(c). Note that there is one major channel and a few minor ones, the major one being shown in Fig. S2(d) with its spine structure. With a larger probe of radius 2.5Å, the channel becomes as in Fig. S2(e), with a simpler structure. Note that when the probe radius is increased to 3.0Å the channel becomes a void within the protein strcucture, having no exit to the outside. We emphasize here that all computations are done with an identical quasi-triangulation of the input structure. In other words, given the 2.3Å channels, the marginal computation time necessary to get 2.5Å channels can be small. The voids and channels in these figures are visualized from the beta-shape representation.

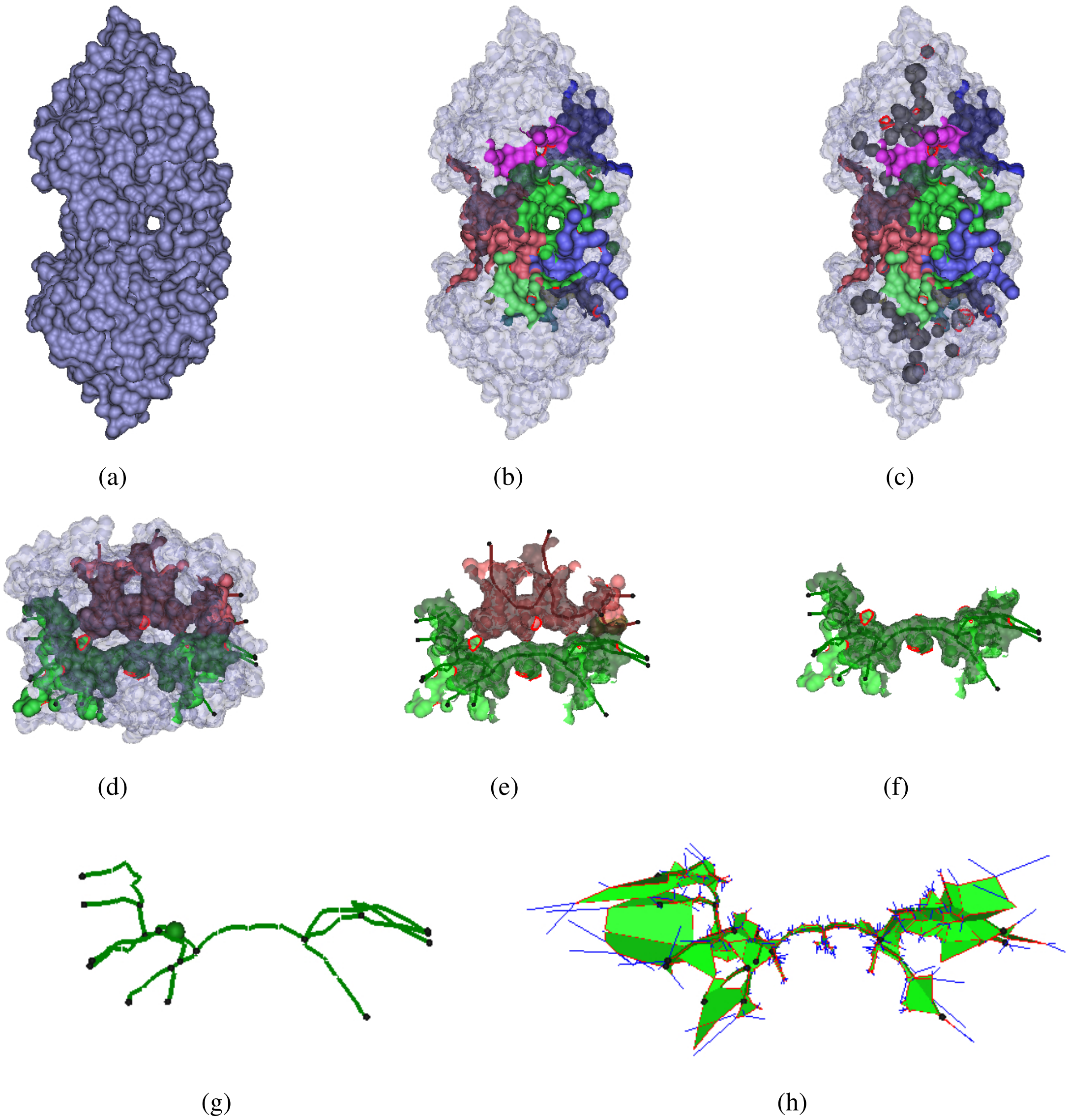

Figure S1: The channels extracted from human carbonic anhydrase XII (PDB code: 1jd0). (a) The Connolly surface with respect to water molecule (probe radius 1.4Å), (b) the recognized channels, (c) the channels plus voids, (d) two major channels from a different view, (e) two major channels with their spines, (f) the biggest channel with its spine, (g) the spine of the biggest channel and its bottleneck ball, and (h) the Voronoi complement for the largest channel.

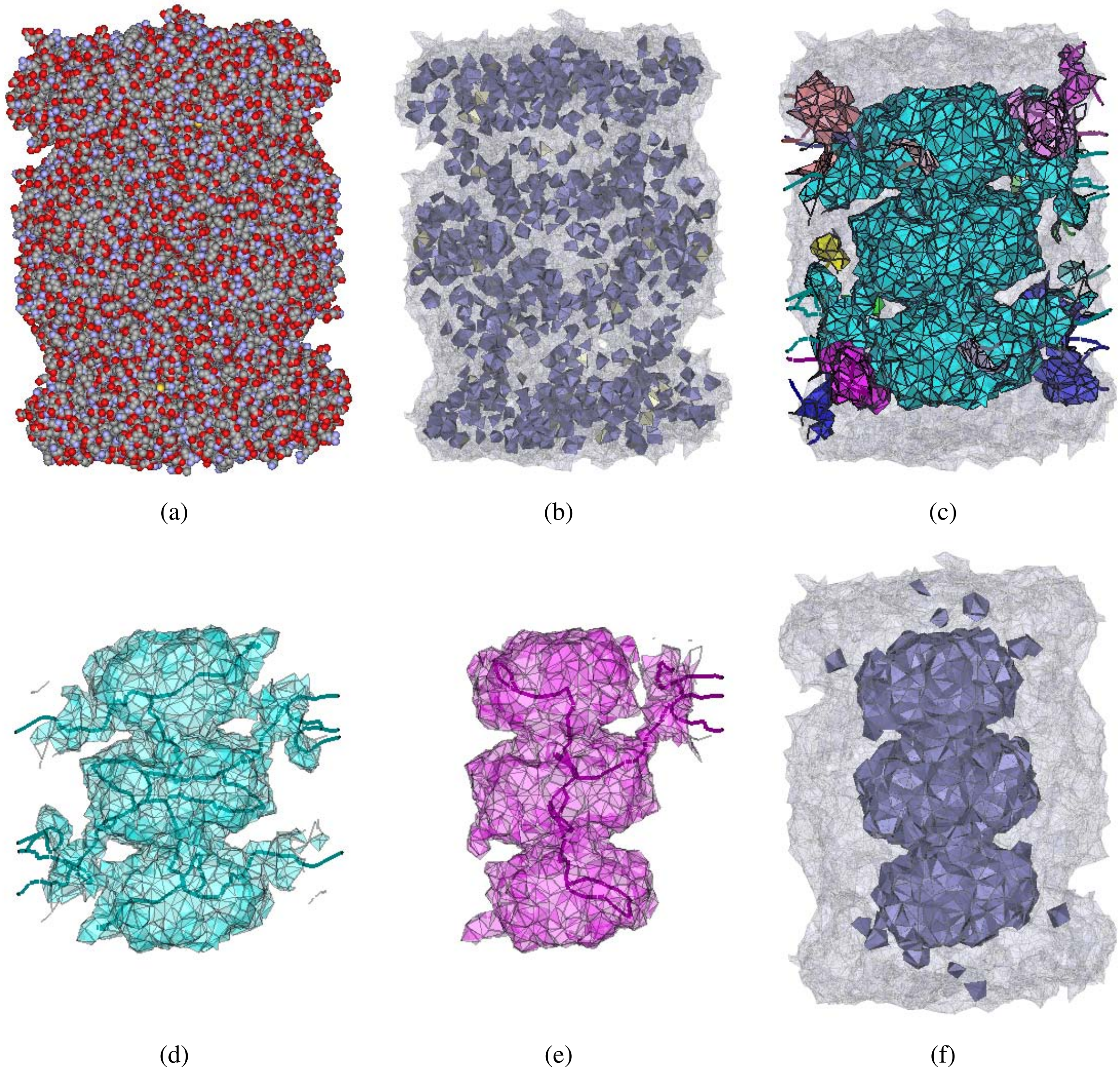

Figure S2: Voids and channels of a proteasome structure obtained using probes of different sizes. (a) The molecule (PDB code: 3shj, # atoms: 50,877) in space-filling representation, (b) voids for water molecules (probe radius: 1.4Å), (c) channels for probe radius 2.3Å, (d) the biggest channel and its spine (thick lines), (e) the biggest channel for probe radius 2.5Å, and (f) voids for probe radius 3.0Å.
